# Supplementary material for: Single-mitosis dissection of acute and chronic DNA mutagenesis and repair
Source: Nat Genet. 2024 Apr 16;56(5):913–24. doi: 10.1038/s41588-024-01712-y (PMC11096113; doi:10.1038/s41588-024-01712-y)
Supplement: Supplementary file 1 — Reporting Summary [file 41588_2024_1712_MOESM1_ESM.pdf]

nature portfolio

Corresponding author(s): Duncan T. Odom
Martin S. Taylor

Last updated by author(s): Mar 6, 2024

# Reporting Summary

Nature Portfolio wishes to improve the reproducibility of the work that we publish. This form provides structure for consistency and transparency in reporting. For further information on Nature Portfolio policies, see our Editorial Policies and the Editorial Policy Checklist.

## Statistics

For all statistical analyses, confirm that the following items are present in the figure legend, table legend, main text, or Methods section.

| n/a | Confirmed | |
|---|---|---|
| ☐ | ☒ | The exact sample size (*n*) for each experimental group/condition, given as a discrete number and unit of measurement |
| ☐ | ☒ | A statement on whether measurements were taken from distinct samples or whether the same sample was measured repeatedly |
| ☐ | ☒ | The statistical test(s) used AND whether they are one- or two-sided<br>*Only common tests should be described solely by name; describe more complex techniques in the Methods section.* |
| ☒ | ☐ | A description of all covariates tested |
| ☐ | ☒ | A description of any assumptions or corrections, such as tests of normality and adjustment for multiple comparisons |
| ☐ | ☒ | A full description of the statistical parameters including central tendency (e.g. means) or other basic estimates (e.g. regression coefficient) AND variation (e.g. standard deviation) or associated estimates of uncertainty (e.g. confidence intervals) |
| ☐ | ☒ | For null hypothesis testing, the test statistic (e.g. *F*, *t*, *r*) with confidence intervals, effect sizes, degrees of freedom and *P* value noted<br>*Give P values as exact values whenever suitable.* |
| ☒ | ☐ | For Bayesian analysis, information on the choice of priors and Markov chain Monte Carlo settings |
| ☒ | ☐ | For hierarchical and complex designs, identification of the appropriate level for tests and full reporting of outcomes |
| ☐ | ☒ | Estimates of effect sizes (e.g. Cohen's *d*, Pearson's *r*), indicating how they were calculated |

*Our web collection on statistics for biologists contains articles on many of the points above.*

## Software and code

Policy information about availability of computer code

| | |
|---|---|
| Data collection | Data was collected using Illumina NovaSeq 6000 and NextSeq 2000 platforms |
| Data analysis | R scripts to reproduce all main figure panels can be downloaded from GitHub (https://github.com/odomlab2/Single-Mitosis-LSE) with DOI 10.5281/zenodo.10786189.<br><br>R packages used in analysis (R version 4.3.0)<br>Python 3.6.1<br>samtools 1.12<br>SNPsplit 0.4.0<br>TrimGalore 0.6.6<br>bowtie2 2.3.5.1<br>Strelka2 2.8.4<br>bcftools 1.10.2<br>gatk-tools 0.2.2<br>bedtools 2.24.0<br>macs2 2.1.2.1<br>Kallisto 0.46.0<br>GenomicRanges 1.52.1<br>Rsamtools 2.12.0<br>randtests 1.0.1<br>Repitools 1.46.0 |

changepoint 2.2.4
RColorBrewer 1.1-3
pheatmap 1.0.12
NMF 0.26
TxDb.Mmusculus.UCSC.mm10.knownGene 3.10.0
BSgenome.Mmusculus.UCSC.mm10 1.4.3
rtracklayer 1.60.0
ggplot2 3.4.4
vcfR 1.14.0
scales 1.2.1
regioneR 1.32.0
Gviz 1.44.2
bsub 1.1.2
viridis 0.6.4
QuasR 1.40.1
maptools 1.1-8
apcluster 1.4.11
mixtools 2.0.0
lsa 0.73.3

For manuscripts utilizing custom algorithms or software that are central to the research but not yet described in published literature, software must be made available to editors and reviewers. We strongly encourage code deposition in a community repository (e.g. GitHub). See the Nature Portfolio guidelines for submitting code & software for further information.

## Data

Policy information about availability of data

All manuscripts must include a data availability statement. This statement should provide the following information, where applicable:

- Accession codes, unique identifiers, or web links for publicly available datasets
- A description of any restrictions on data availability
- For clinical datasets or third party data, please ensure that the statement adheres to our policy

Fastq files for the WGS, RNA and ATAC-seq described here can be downloaded from Sequence Read Archive (SRA) under the accession number PRJNA934746. Processed files including mutation calls, TPM counts and ATAC peaks used in the analysis have been deposited in GEO under the accession GSE230579.

## Research involving human participants, their data, or biological material

Policy information about studies with human participants or human data. See also policy information about sex, gender (identity/presentation), and sexual orientation and race, ethnicity and racism.

| | |
|---|---|
| Reporting on sex and gender | N/A |
| Reporting on race, ethnicity, or other socially relevant groupings | N/A |
| Population characteristics | N/A |
| Recruitment | N/A |
| Ethics oversight | N/A |

Note that full information on the approval of the study protocol must also be provided in the manuscript.

# Field-specific reporting

Please select the one below that is the best fit for your research. If you are not sure, read the appropriate sections before making your selection.

☒ Life sciences ☐ Behavioural & social sciences ☐ Ecological, evolutionary & environmental sciences

For a reference copy of the document with all sections, see nature.com/documents/nr-reporting-summary-flat.pdf

# Life sciences study design

All studies must disclose on these points even when the disclosure is negative.

| | |
|---|---|
| Sample size | A minimum of 5 pairs was desired to assess reproducibility and increase total mutation numbers to study. 7 replicates were conducted in case some samples were problematic. No specific calculation was performed for mouse tumour selection. Instead, we sought to investigate 3 |

| | |
|---|---|
| | unique tumours from 2 independent mice (6 in total) to ascertain haplotype resolution reproducibility between both tumours and mice. |
| Data exclusions | No data were excluded. |
| Replication | General mutation patterns were interrogated across all replicates, both in cell culture and tumours, and each figure displays points for each replicate for the corresponding contrast. |
| Randomization | Randomization of sister pairs and mice was not relevant as this is not a case-controlled study. |
| Blinding | Blinding was not relevant as these experiments were not a case-controlled study. |

# Reporting for specific materials, systems and methods

We require information from authors about some types of materials, experimental systems and methods used in many studies. Here, indicate whether each material, system or method listed is relevant to your study. If you are not sure if a list item applies to your research, read the appropriate section before selecting a response.

## Materials & experimental systems

| n/a | Involved in the study |
|---|---|
| ☒ | ☐ Antibodies |
| ☐ | ☒ Eukaryotic cell lines |
| ☒ | ☐ Palaeontology and archaeology |
| ☐ | ☒ Animals and other organisms |
| ☒ | ☐ Clinical data |
| ☒ | ☐ Dual use research of concern |
| ☒ | ☐ Plants |

## Methods

| n/a | Involved in the study |
|---|---|
| ☒ | ☐ ChIP-seq |
| ☐ | ☒ Flow cytometry |
| ☒ | ☐ MRI-based neuroimaging |

# Eukaryotic cell lines

Policy information about cell lines and Sex and Gender in Research

| | |
|---|---|
| Cell line source(s) | P388D1 cells from ATCC and Lentix HEK293T |
| Authentication | No authentication was carried out. |
| Mycoplasma contamination | Cell lines tested negative for mycoplasma contamination. |
| Commonly misidentified lines (See ICLAC register) | No commonly misidentified cell lines was used in this study. |

# Animals and other research organisms

Policy information about studies involving animals; ARRIVE guidelines recommended for reporting animal research, and Sex and Gender in Research

| | |
|---|---|
| Laboratory animals | Inbred female Mus musculus castaneus (CAST/EiJ) mice were crossed with inbred male C3H/HeOuJ (C3H) mice. The F1 offspring were treated with a single intraperitoneal dose of N-Nitrosodiethylamine (DEN; Sigma-Aldrich N0258; 20 mg/kg body weight) at P15. Liver tumours were isolated 30 weeks after treatment and stored at -80°C for DNA extraction and sequencing. Liver tissue from an untreated P15 litter mate was sampled for control experiments. Control samples (liver tissue) were also collected from untreated, age-matched littermates. Animals were maintained using standard husbandry: mice were group housed in Tecniplast GM500 IVC cages at room temperature/humidity with a 12 h:12 h light:dark cycle and ad libitum access to water, food (LabDiet 5058), and environmental enrichments. |
| Wild animals | This study did not use wild animals. |
| Reporting on sex | Only males were used, as females are relatively resistant to DEN induced tumorigenesis. |
| Field-collected samples | This study did not use field collected samples. |
| Ethics oversight | Animal experimentation was carried out in accordance with the Animals (Scientific Procedures) Act 1986 (United Kingdom) and with the approval of the Cancer Research UK Cambridge Institute Animal Welfare and Ethical Review Body (AWERB). |

Note that full information on the approval of the study protocol must also be provided in the manuscript.

# Flow Cytometry

## Plots

Confirm that:

☒ The axis labels state the marker and fluorochrome used (e.g. CD4-FITC).

☒ The axis scales are clearly visible. Include numbers along axes only for bottom left plot of group (a 'group' is an analysis of identical markers).

☒ All plots are contour plots with outliers or pseudocolor plots.

☒ A numerical value for number of cells or percentage (with statistics) is provided.

## Methodology

| | |
|---|---|
| Sample preparation | To ascertain DNA content, 2 drops of Hoechst 33342 Ready Flow™ Reagent from Invitrogen™ was added to 2 x 106 cells and placed in the incubator for 15 minutes. Cells were spun down, resuspended in Miltenyi Biotec FACs buffer and assayed using the BD FACSAria™ Fusion 3 system. Green fluorophores were ascertained with excitation at 488 nM and emission at 530 nM, while the orange fluorophore of G1 cells was excited at 561 nM and emission recorded at 586 nM. |
| Instrument | BD FACSAria Fusion 3 |
| Software | BD FACSDiva software 8.0.2 |
| Cell population abundance | Histogram of Hoechst staining of DNA content in supplemental figure 1c shows all signal for gated cells as depicted in supplemental gating figure. For single cell sorting to create the PF1 clone, positive Green fluorescence using signal as depicted in the supplemental gating figure was used. |
| Gating strategy | Live cells were determined by FSC-A and SSC-A, and singlets gated with FSC-A and FSC-H. Cells with positive emission in the 530/30 after excitation with a blue laser at 488nM were sorted as singlets for clonal selection. |

☒ Tick this box to confirm that a figure exemplifying the gating strategy is provided in the Supplementary Information.
